# Essential maternal health service disruptions in Ethiopia during COVID 19 pandemic: a systematic review

**DOI:** 10.1186/s12905-022-02091-4

**Published:** 2022-12-06

**Authors:** Amare Zewdie, Ayenew Mose, Ali Yimer, Tamirat Melis, Ahmed Nuru Muhamed, Abdurezak Kemal Jemal

**Affiliations:** 1 Department of Public Health, College of Medicine and Health Science, Wolkite University, Wolkite, Ethiopia; 2 Department of Midwifery, College of Medicine and Health Science, Wolkite University, Wolkite, Ethiopia; 3 Department of Public Health, College of Medicine and Health Science, Weldia University, Weldia, Ethiopia; 4 Department of Nursing, College of Medicine and Health Science, Wolkite University, Wolkite, Ethiopia

**Keywords:** Impact of COVID 19, Essential maternal health service, Systematic review, Ethiopia

## Abstract

**Introduction:**

COVID 19 pandemic has challenged the resilience of the most effective health systems in the world. The Ethiopian Ministry of health tried to ensure the continuation of essential maternal health services during the pandemic. Despite several individual studies conducted on the impact of COVID 19 on maternal health services, no evidence can summarize the extent of impact as a nation and which essential maternal health service is most affected.

**Method:**

A systematic review was conducted to summarize the extent of disruption of essential maternal health services and identify the most affected service in the era of the COVID pandemic in Ethiopia. Preferred Reporting Items for Systematic Review and Meta-analysis guidelines were followed. Comprehensive literature was searched using international databases PubMed, Google scholar, and African Online Journal to retrieve related articles. Descriptive analysis was made to answer the review objective.

**Result:**

Overall, 498 articles were retrieved using our search strategy and finally 8 articles were included in the review. We found, ANC (26.35%), skilled birth attendance (23.46%), PNC (30%), family planning (14%), and abortion care (23.7%) maximum disruption of service utilization due to the pandemic. PNC service was the most significantly affected service unit followed by the ANC unit.

**Conclusion:**

Essential maternal health services have been significantly disrupted due to COVID 19 pandemic in Ethiopia. It is expected from all stakeholders to prioritize safe and accessible maternity care during the pandemic and the aftermath and take lesson to reduce maternal and infant morbidity and mortality.

**Supplementary Information:**

The online version contains supplementary material available at 10.1186/s12905-022-02091-4.

## Introduction

Well-arranged and prepared health systems can endure to provide equitable access to basic and essential services throughout an emergency [1]. When health systems are restrained, direct mortality from the pandemic and indirect mortality from preventable and treatable conditions increase significantly. Analyses from the 2014–2015 Ebola outbreak suggest that deaths resulting from malaria, measles, tuberculosis (TB), HIV/AIDS, and health system failures outweighed deaths from Ebola [2–4]. COVID 19 pandemic has challenged the resilience of the most effective health systems in the world. In many settings, health care service provision had been modified to focus on managing COVID-19 cases, and this approach has been affecting the delivery of essential health services including reproductive, maternal, neonatal, and child health services [5]. A modeling study of essential maternal and child health interventions across 118 low- and middle-income countries over 6 months estimated a reduction of services by 9.8–18.5% and 39.3–51.9% in the least and most severe scenarios, respectively, due to the pandemic [6].

The already fragile health system in most countries in Africa is overburdened with the additional mission to address the COVID 19 pandemic which minimizes its ability to provide essential health services [7]. Essential maternal health services include antenatal care, skilled birth attendance (SBA), postnatal care (PNC), and other sexual and reproductive health care services such as family planning (FP), post-abortion care, and, where legal, safe abortion services [8]. The WHO recommends the continuation of essential maternal health services regardless of COVID 19 expansion which shows the inevitability of those services [9]. However, many countries, particularly countries in Africa including Ethiopia have faced substantial challenges to maintain the provision of high-quality essential maternal health services in the era of the COVID pandemic [10].

Ethiopia reported the first COVID 19 case on March 13, 2020, and the pandemic is causing several cases and deaths [11]. Following the WHO advice after confirming the first case, the Ethiopian federal ministry of health (MOH) initiated different prevention strategies such as a partial and selective lockdown, the prohibition of mass gatherings such as mass praying, and the closure of schools. At the same time, the MOH ensured the continuation of essential services, such as reproductive, maternal, neonatal, child, and adolescent health services, and the management of major communicable and non-communicable diseases, surgical care, and emergency and critical care [12]. Despite several studies conducted on the impact of COVID 19 on maternal health services in different regions of the country [13–20], there is a lack of evidence that can recapitulate the extent of impact as a nation and which essential maternal health service is most affected. Thus this systematic review aims to summarize the extent of disruption of essential maternal health services and identify the most significantly affected service in the era of the COVID pandemic in Ethiopia.

### Significance of the study

This review can represent the Ethiopian context and it could help policymakers effectively manage the indirect effects of COVID 19 on maternal and child health. Findings from this review will be important for all stakeholders at all levels of healthcare systems in providing necessary information which might be used to develop a new strategy for the alleviation of the negative impact of COVID 19 on the utilization of essential maternal health services and its consequences. Furthermore, the review identifies the most significantly affected unit of essential maternal health service; it will inform the respective stakeholder to strategically direct to improve the respective service.

## Method

### Study design and setting

A systematic review was conducted to summarize the extent of disruption of essential maternal health services and identify the most affected essential maternal health service in the era of the COVID pandemic in Ethiopia. Ethiopia is one of the low-income countries located in the horn of Africa with a projected population of 133.5 million in 2032 and 171.8 million in 2050 [21]. For administrative purposes, Ethiopia is divided into 11 regions and 2 city administrations. Regions are further classified into zones and zones are divided into districts. Finally, districts are divided into kebele (the smallest administrative division contains 2000 up to 2500 residents). Preferred Reporting Items for Systematic Review and Meta-Analysis guidelines were used for this review (Additional file 1: Table S1). PRISMA is a protocol consisting of checklists that guide the conduct and reporting of systematic reviews and meta-analyses, which increase the transparency and accuracy of reviews in medicine and other fields [22].

### Search strategies and sources of information

We have checked the PROSPERO database (http://www.library.ucsf.edu/) and the resources on COVID-END (COVID-END) whether published or ongoing projects exist related to the topic to avoid any further duplication. Thus, the findings revealed that there were no ongoing or published articles in the area of this topic. Then this systematic review was registered in the PROSPERO database with Id no of CRD42022322284. Comprehensive literature was searched using international databases PubMed, Google scholar, and African Online Journal to retrieve related articles from March 22 to 30, 2022. Grey literature was searched using Google. Search terms were formulated using PICO guidelines through the online databases. Medical Subject Headings (MeSH) and key terms had been developed using different Boolean operators ‘AND’ and ‘OR’. The following search term was used: “impact of COVID 19” OR impact of SARS-CoV-2” OR “effect of COVID 19” OR “effect of SARS-CoV-2” AND “essential maternal health service” OR “basic maternal health service” OR “maternal health service” OR “health service” AND Ethiopia.

### Eligibility criteria

In this systematic review, we include studies that meet the following criteria. Firstly, the study must be done on the impact of COVID 19 on maternal health services. Secondly, no restriction was made regarding population group, race, and publication date but to the last search date that is March 30, 2022. Studies that employ a qualitative method and do not report the proportion of service disrupted, studies with data extracted, duplicate, abstract-only papers, articles without available full text, conference, editorial, case reports, case series, and systematic review studies are excluded at each respective stage of screening.

### Outcome measurements

In this review, the outcome of interest was the proportion of essential maternal health services disrupted by the COVID 19 pandemic. It is defined as the proportion of service which is not delivered as a result of the expansion of the virus. Therefore, all included studies were reporting pre-COVID 19-period service utilization and comparison pandemic-period service utilization. Then the proportion of service disrupted was calculated. Finally, the most significantly affected unit of essential maternal health service that needs major focus while we design intervention to improve utilization of those services was identified.

### Data extraction

All studies obtained from all databases were exported to Endnote version X8 software to remove duplicate studies. Then after, all studies were exported to a Microsoft Excel spreadsheet. The authors independently extracted all the important data using a standardized data extraction form which was adapted from the Joanna Briggs Institute (JBI) data extraction format. The data extraction format included (primary author, year of publication, region, study design and pre and post COVID service utilization in each department of maternal health service.

### Quality assessment

To assess the quality of each study included in this systematic review, the modified Newcastle Ottawa Quality Assessment Scale for cross-sectional studies was used [23]. Each Author has assessed the quality of each study (i.e. methodological quality, sample selection, sample size, comparability and the outcome, and statistical analysis of the study) (Additional file 2: Table S2). In the case of disagreement between authors; another author was involved and discussed and resolved the disagreement.

### Data processing and analysis

Selected articles were entered into Microsoft Excel spreadsheet format for analysis. For each study, service utilization in the pre-COVID 19 and during the COVID 19 period was extracted. To achieve our objective, essential maternal health service disruption, we calculated service disruption considering the pre and COVID pandemic period data. Service disruption for each study was calculated as; service utilization during the COVID pandemic minus service utilization in pre COVID period multiplied by 100. Then to summarize the evidence that we get from the included studies descriptive analysis such as the maximum disruption of each essential maternal health service was made. Then identification of the most significantly affected unit of essential maternal health service that needs focus was made for possible use in a practical setting.

### Patient and public involvement

In this review, neither patient nor the public was involved in the study design, conduct, reporting, or dissemination plans of our research.

## Result

**Fig. 1:**
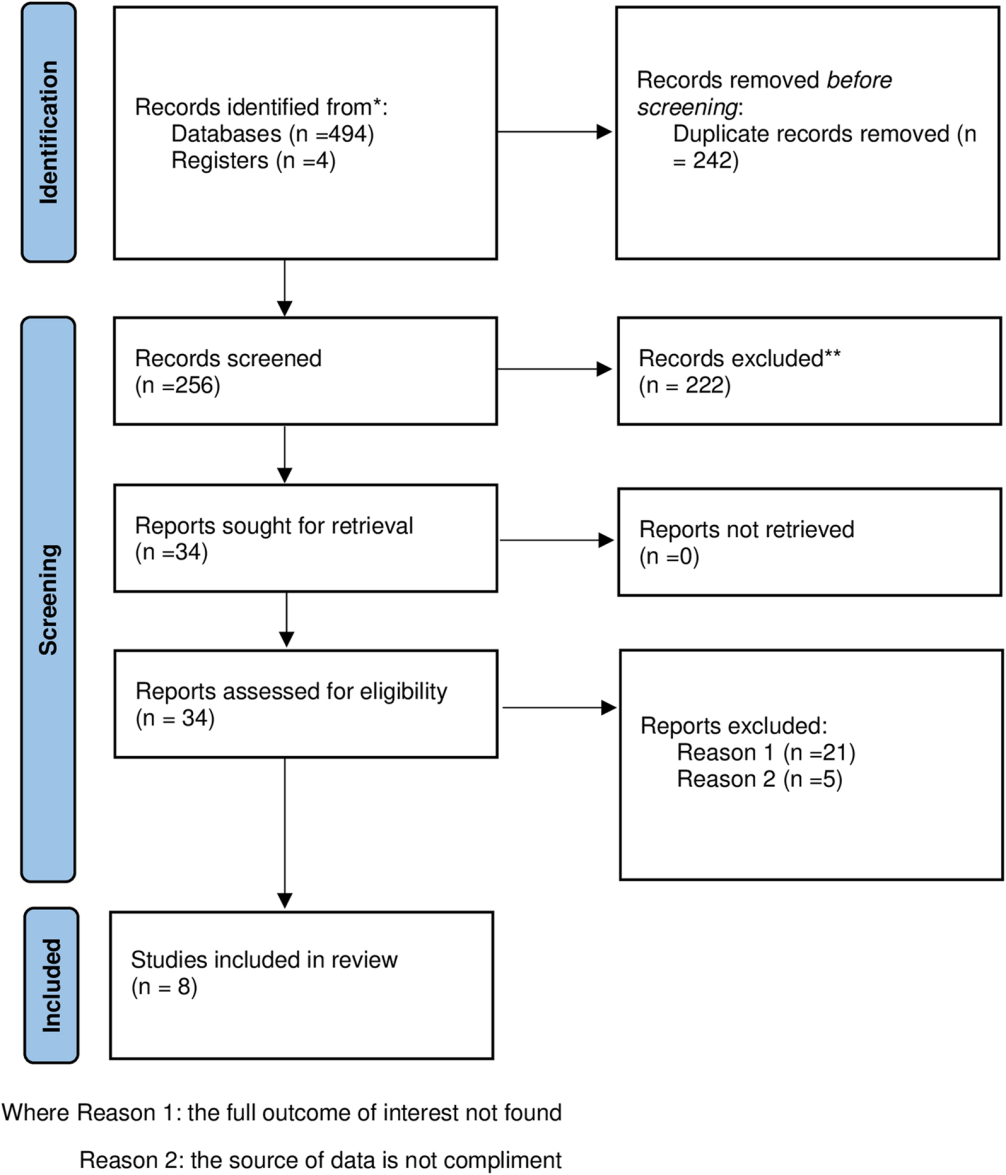
PRISMA 2020 flow diagram followed for the systematic review

Overall, 498 articles were retrieved using a search strategy about the impact of COVID 19 on essential maternal health services. International databases; PubMed, Google scholar, and African Journals Online were searched. Duplicates (242) were removed and 256 articles remained. After reviewing, (n = 119) articles were excluded by title, and (n = 103) articles were excluded by reading abstracts. Therefore, 34 full-text articles were accessed and assessed for inclusion criteria, resulting in the further exclusion of 26 Articles primarily due to reasons. As a result, 8 studies that fulfilled the inclusion criteria undergo the final systematic review (Fig. 1).

All included studies report two or more maternal health service units’ utilization status and most of the studies documented the utilization status of the five essential maternal health services. Of the reviewed articles one was done nationally and 3, 1,1,1,1were done at Addis Ababa, Amhara, Tigray, Diredawa, and south nation nationality and people region (SNNPR) respectively. Regarding comparison period; all included studies use equivalent time-period in the pre-COVID and COVID 19 periods. Accordingly among the included studies; 4 studies use average monthly service utilization whereas, the remaining use annual, 8, 4, and 3-months data (Table 1).

**Table 1:** study characteristics and proportion of essential maternal health services disrupted due to COVID 19 in Ethiopia, 2022

Primary author	Year of publication	Region	Length of period for comparison	Percentage of essential maternal health services disrupted due to COVID 19					Study quality score
				ANC%	SBA %	PNC %	FP %	AC%	
Shuka et al. [13]	2021	National	Average monthly	− 9.19	ND	ND	− 7.60	ND	8
Bekele et al. [14]	2022	Amhara	Average monthly	− 13.02	ND	ND	ND	ND	8
Ayele et al. [15]	2021	Addis Ababa	Average monthly	− 8.10	− 9.80	NI	NI	NI	7
Tefera et al. [16]	2022	Diredawa	Annual	ND	ND	− 30.0	ND	NI	8
Kassie et al. [17]	2021	SNNPR	4 months	− 26.35	− 23.46	− 29.1	ND	ND	9
Desta et al. [18]	2021	Tigray	3 months	− 2.83	ND	ND	− 4.81	− 12.31	8
Gebreegziabher et al. [19]	2022	Addis Ababa	8 months	− 7.04	− 2.50	− 9.3	− 20.5	− 23.7	9
Enbiale et al. [20]	2021	Addis Ababa	Average monthly	ND	ND	NI	− 14.0	NI	7
Maximum disruption				− 26.35	− 23.46	− 30.0	− 14.0	− 23.7	

*ANC* antenatal care, *SBA* skilled birth attendance, *PNC* postnatal care, *FP* family planning, *AC* abortion care, *ND* not disrupted, *NI* not included in the study

## Essential maternal health service disruption due to COVID 19 in Ethiopia

### ANC service disruption

From included studies, ANC service was disrupted in 6 of the studies even if there was no ANC service disruption reported in 2 studies. Maximum ANC service disruption was calculated as 26.35% in the study done at SNNPR followed by 9.8% in Addis Ababa. It was the second essential maternal health service unit in which the largest service disruption was recorded next to the PNC service unit.

### Skilled birth attendance (SBA) or delivery service disruption

Delivery service was disrupted in 3 studies and not disrupted in 5 studies. Maximum Skilled birth attendance service disruption was computed as 23.46% in the study done at SNNPR followed by 13.02% in the Amhara region.

### PNC service disruption

PNC service disruption was detected in three studies. The maximum PNC service disruption was calculated as 30% in the study done at Diredewa followed by 29.1% in SNNPR. PNC service was the unit that was most significantly affected by COVID 19 pandemic since it has the largest essential maternal health service disruption in this review.

### Family planning service disruption

Family planning service was disrupted in half [4] of the studies even if it showed no disruption in half of the studies. Maximum Family planning service disruption was calculated as 14.0% in the study done at Addis Ababa followed by 4.81% in the Tigray region.

### Abortion care service disruption

Among the included studies abortion care service disruption was reported in two studies. The maximum abortion care service disruption was calculated as 23.7% in the study done at Addis Ababa followed by 12.31% in Tigray.

## Discussion

Humanitarian emergencies, such as pandemics and disasters, cause an unprecedented disruption in the provision of routine health services. Moreover, the outbreak of COVID 19 which is declared a global Public Health Emergency, disrupted healthcare services of most vulnerable communities, such as pregnant women and children [24]. Ethiopia is one of the Sub-Saharan African countries which are severely affected by the COVID 19 pandemic. The pandemic imposed direct and indirect health consequences on the population of the country including service delivery and utilization [25]. The lack of essential health conditions leads to maternal and infant morbidity and mortality. In the context of already poor health outcomes, significant reductions in service utilization for maternal and child health may have substantial adverse impacts [26]. A modeling study of 118 low-and middle-income countries estimated an additional 12,200–56,700 maternal and 253,500–1,157,000 child deaths using several hypothetical scenarios in which the coverage of essential maternal and child health services was reduced by 9.8–51.9% due to the pandemic over 6 months [6].

Ensuring the resilience of essential maternal health services is crucial in reducing the direct and indirect impacts of the pandemic. This systematic review was aimed to assess the magnitude of essential maternal health service disruption in Ethiopia. Accordingly, there was significant essential maternal health service disruption in every five departments of the maternal health service unit in the country. This is supported by evidence from different sources, where the majority of health services were disrupted, and where essential health services like MCH were highly affected due to COVID 19 presence [27]. This may be attributable to fear and anxiety about the new pandemic due to social and mainstream media predisposition, nationwide impacted prevention protocols limiting movements and closure of some facilities, transport system disruption, as well as the health system resilience in low and resource-limited countries like Ethiopia. This disruption may negatively affect the health of mothers and children which may contribute to increment in avoidable maternal and child death. This evidence suggests that focus should be made to social and mainstream media communication during pandemic like COVID 19 to minimize excessive fear which diminish service utilization.

Our review found a maximum of 26.35% ANC service disruption in Ethiopia due to COVID 19 pandemic. This finding was comparable with national evidence of Haiti, Sierra Leone, Mozambique, and India in which ANC service utilization showed an 18%, 32%, 26%, and 22.91% decline respectively from March to December 2020 as compared to the expected service utilization [28–30]. The finding is also consistent with the Nepal, Rwanda, and Kenya studies where a fall in ANC service was observed in the first 2 months of the COVID pandemic [31–33]. This might be related to an inadequate supply of personal protective equipment, redirecting the human workforce and services toward responding to the COVID19 pandemic. In addition, fear of acquiring COVID-19 by clients, and financial barriers of seeking health services might negatively affect services utilization [34]. This implies that there was a high proportion of missed mothers, who were supposed to follow ANC and gain important service during their ANC follow-up. Since counsiling and health service which would be given during ANC follow up are basic and necessary for maternal continuum of care including facility delivery and postnatal care. The finding implies effort should be made to compensate for the disrupted health service and to increase ANC service utilization so as to minimize the consequences of ANC service disruption.

In our review, the maximum skilled birth attendance service disruption due to the COVID pandemic was 23.46%. The finding was supported by evidence from different countries across the globe such as Rwanda, Haiti, Mozambique, Liberia, Lesotho, Sierra Leone, India, and Mexico [28–30, 32]. Where the evidence had shown a significant fall in skilled birth attendant service during the time of COVID 19 in their respective countries. The fall in facility delivery could be attributed to COVID 19 announcement and related social and family reinforcements, ANC service decrement, and available strong social values encouraging home deliveries might be pronounced by the poor health system resilience to maintain mothers in the service continuum. The finding implies respective stakeholders should address the gap in facility delivery service utilization and try to resilience and strength their effort to make a remedy to the victims of service disruption.

This review identifies 30% maximum PNC services disruption in Ethiopia. Again PNC service was the unit that was most significantly affected by the COVID 19 pandemic since it has the largest proportion of service disruption. Studies that are done in sub-Saharan African countries, Rwanda, and SouthAfrica had similar findings that postnatal care services had shown a significant decrease following the months of the COVID 19 announcement [32, 35, 36]. The routine trend, where PNC is not considered as part of the routine delivery care in addition to the existing anxiety about the global pandemic, disruption of ANC and delivery service by the pandemic, and social distortions may be considered as the pressing factors for the decreased PNC service utilization. As postnatal period is critical time which contributes largest proportion for maternal death which implies resilence of this essential health service is crucial. This evidence suggests we should strengthen our effort to resilence the PNC service utilization so as to minimize maternal and infant morbidity and mortality in the postpartum period and thereafter.

Maximum Family planning service disruption in Ethiopia due to the COVID pandemic found to be 14.0%. This finding is congruent with other studies where a fall in Family planning services was observed in Bangladesh, South Africa, Nigeria [37], and Mozambique [30]. Where the fall is attributable to similar reasons that people were suffering from fear and anxiety about the disease and multifaceted protective measures taken by local authorities, that had limited movements. However, another study has shown an increase in short-term and injectable family planning utilization by adolescents during the time [31]. The reason for the discrepancy might be adolescents were exposed to sexual practice, abuse, and violence due to the lockdown and school closures. Additionally, our review is not restricted to short-term contraceptives and adolescents but rather to all contraceptive methods and all age groups.

Finally, in our review, we have found that the maximum abortion care service disruption was 23.7%. this finding is supported by a study done in Louisiana in which the number of abortion care Louisiana residents decreased by 31% [38]. However, the finding is against from study done in Kenya [31] in which abortion care services were increased during the COVID19 pandemic. The increased post-abortion care might be explained by women, who stayed at home during the lockdown and were more prone to sexual abuse including rape even by their partners, and the decrement in family planning use may be another reason. The finding implies effort should be made to minimize the negative effect of lockdown on women health including sexual abuse and rape which result in unsafe abortion and its severe complication.

Not standing with its finding, this systematic review has limitations. First, there were limited studies to include [8] in this systematic review. Our search strategy found limited studies especially no studies from Afar, Gambella, Sidama, Hariri, Somali and Benishangul-Gumuz regions and this calls into question the national representativeness of our study. Furthermore, the included studies were done by reviewing health service data which has the drawback of incompleteness so, this review also shares limitations of the primary studies.

## Conclusion

While rigorous individual studies have been conducted, evidence from this summarized systematic review shows that essential maternal health services are significantly affected as a consequence of the COVID 19 pandemic. Despite commendable efforts made in maintaining several of the essential maternal health services, COVID 19 has led to a decrement in ANC, skilled birth attendance, PNC, family planning, and abortion care in Ethiopia. PNC service utilization was the most significantly affected unit of essential maternal health service. As a result, it is expected from all stakeholders to prioritize safe and accessible maternity care during the pandemic and the aftermath, while planning for a future of radically inclusive and accessible maternity care that will draw on the lessons of this pandemic to reduce preterm birth, stillbirth, and maternal mortality nationally. Hence, the health system should mobilize the workforce and direct its approach to community outreach service to access mothers in need of essential maternal health services.

## Supplementary Information

**Additional file 1**. **Table S1**: PRISMA 2020 Checklist which used In the systematic review 2022.

**Additional file 2**. **Table S2**: Newcastle-Ottawa Quality Assessment Scale for cross sectional studies used in the systematic review 2022.

## Data Availability

The result of this systematic review was extracted from the data gathered and analyzed based on the stated methods and materials. All the relevant data are within the paper.

## Abbreviations

ANC: Antenatal care
COVID 19: Coronavirus disease 2019
PNC: Postnatal care
PRISMA: Preferred Reporting Items for Systematic Reviews and Meta-Analyses
