# Supplementary material for: Essential maternal health service disruptions in Ethiopia during COVID 19 pandemic: a systematic review
Source: BMC Womens Health. 2022 Dec 6;22:496. doi: 10.1186/s12905-022-02091-4 (PMC9724383; doi:10.1186/s12905-022-02091-4)
Supplement: Supplementary file 2 — Additional file 2. Table S2: Newcastle-Ottawa Quality Assessment Scale for cross sectional studies used in the systematic review 2022. [file 12905_2022_2091_MOESM2_ESM.docx]

| **Supplementary table 2 :** Newcastle-Ottawa Quality Assessment Scale for cross sectional studies **used in the systematic review 2022** | | | | | | | | |
| --- | --- | --- | --- | --- | --- | --- | --- | --- |
|  | Selection | | | | Comparability | Outcome | | Total score |
| Authors | Representativeness s (1) | Sample size (1) | Non respondents (1) | Ascertainment of the exposure (risk factor) (2) | The subjects in different outcome groups are comparable, based on the study design or analysis. confounding factors are controlled (2) | Assessment of the outcome (2) | Statistical test (1) |  |
| Shuka, Zemzem Shigute, et al(13) | 1 | 1 | 1 | 1 | 1 | 2 | 1 | 8 |
| Bekele, Chalachew, et al.(14) | 1 | 1 | 1 | 1 | 2 | 1 | 1 | 8 |
| Ayele, Wondimu, et al.(15) | 1 | 1 | 1 | 1 | 1 | 1 | 1 | 7 |
| Tefera, Bereket, et al.(16) | 1 | 1 | 1 | 1 | 2 | 1 | 1 | 8 |
| Kassie, A., Wale, A. and Yismaw, W.,(17) | 1 | 1 | 1 | 1 | 2 | 2 | 1 | 9 |
| Desta, Abraham Aregay, et al.(18) | 1 | 1 | 1 | 1 | 1 | 2 | 1 | 8 |
| Gebreegziabher, Senedu Bekele, et al.(19) | 1 | 1 | 1 | 1 | 2 | 2 | 1 | 9 |
| Enbiale, Wendemagegn, et al.(20) | 1 | 1 | 1 | 1 | 1 | 1 | 1 | 7 |
